# Operation of Amorphous TiO_2_-Protected Photocathodes Described with the Maxwell Equivalent Circuit

**DOI:** 10.1021/acsami.4c07588

**Published:** 2024-10-22

**Authors:** Erin Service, Thomas Moehl, S. David Tilley

**Affiliations:** † Department of Chemistry, University of Zurich, Zurich 8057, Switzerland

**Keywords:** electrochemical impedance spectroscopy, equivalent circuit, amorphous TiO_2_, photocathode, atomic layer deposition, hydrogen evolution

## Abstract

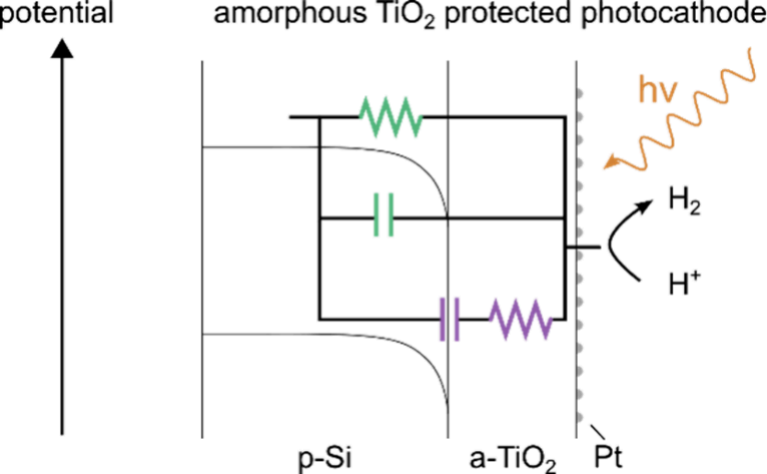

The use of amorphous TiO_2_ (a-TiO_2_), deposited by atomic layer deposition, is a common strategy to protect semiconductors from degradation when used in water-splitting photoelectrochemical (PEC) cells. Electrochemical impedance spectroscopy (EIS) is a suitable technique to study these PEC cells because it is capable of deconvoluting multiple processes occurring during operation, therefore providing information about mechanisms leading to the overall device performance. When biased under hydrogen evolution conditions, EIS shows that two simultaneous processes occur in a-TiO_2_-protected photocathodes, which introduces an ambiguity in choosing the correct equivalent circuit to describe the operating device. In this report, a model p-Si|a-TiO_2_|Pt photocathode system is used to show that the Maxwell circuit best describes the operating mechanism, as opposed to the more commonly used Voight and nested circuits. This indicates that, under hydrogen evolution conditions, both faradaic and nonfaradaic processes are occurring. Whereas the faradaic process corresponds to the hydrogen evolution reaction itself, the nonfaradaic process is traced to the p-Si|a-TiO_2_ interface.

## Introduction

Understanding underlying processes behind the performance of multilayer photoelectrochemical (PEC) devices is crucial to their continued development. As an *operando* technique, electrochemical impedance spectroscopy (EIS) is capable of detecting both processes within individual layers and interactions between the layers themselves during device operation. These devices commonly employ amorphous TiO_2_ (a-TiO_2_) as a protection layer against corrosion, because many established and emerging semiconductors themselves are unstable during photoelectrocatalysis.^1,2^ Here, we resolve the appropriate model to describe operating photocathodes performing the hydrogen evolution reaction (HER) using EIS and propose the origin of a low-frequency process previously observed in the literature.^3,4^

**Figure 1:**
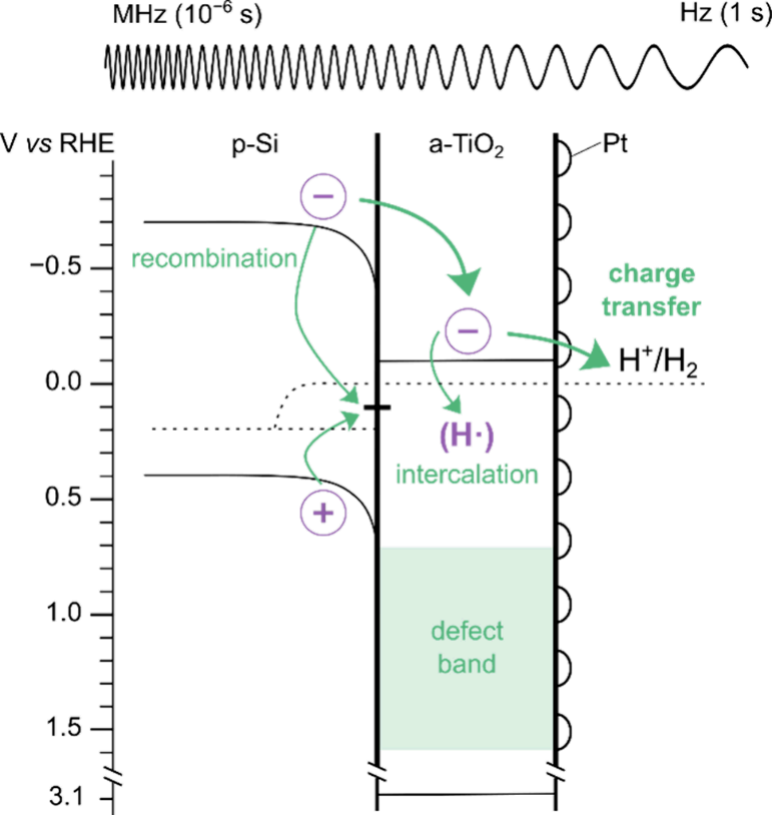
Photocathode device protected by a-TiO_2_, showing possible charge-carrier processes in both the solid-state layers and the electrolyte solution. This figure is not to scale. The p-Si space-charge region width is on the order of 1 μm, while the total width of the Si wafer is 525 μm. The width of a-TiO_2_ is 100 nm. Due to the high doping density of a-TiO_2_, the very narrow space charge width of this material is not shown. In the H_2_-saturated solutions of this study, the buried junction is expected to equilibrate with the H^+^/H_2_ redox couple, as opposed to the work function of Pt in vacuum. The quasi-Fermi energies of the electrons and holes under illumination in p-Si are shown, as well as the Fermi energies of TiO_2_ and the electrolyte. Given the Fermi energies of p-Si and a-TiO_2_, the theoretical quasi-Fermi energy splitting in p-Si is 0.5 V. However, we have drawn the splitting to be 0.2 V, corresponding to the experimental results of this study. The frequency scale at the top of the figure indicates the range of time scales accessible using EIS.

As illustrated in Figure 1, processes in PEC devices are either electronic or ionic in nature. Within the solid state layer(s), electronic processes such as charge pair generation, recombination and carrier diffusion occur, which influence parameters such as the onset potential and photocurrent density. Ionic processes within solids, such as intercalation and ion migration, can also be present.^5−7^ Within the electrolyte solution, electrochemical reactions, ionic diffusion, and reorganization of the Helmholtz layer can occur.^8−10^ The time scales of electronic properties generally range from femtoseconds to microseconds, while those of ionic processes range from microseconds to tens of seconds.^11^ The ability to observe these processes using EIS depends on the time scale of the process. Using common instrumentation, EIS detects processes occurring between microseconds and seconds.

EIS observes these processes under steady-state conditions by monitoring the response of the device to a small voltage perturbation that is superimposed on a dc bias. Resistances and capacitances are extracted by modeling the raw data as an equivalent circuit (EC), the components of which may represent physical processes occurring within the device. Most processes observed for PEC devices (and all discussed here) appear as semicircles in the Nyquist plot. At potentials where EIS detects only one of these processes, the device is modeled with a resistor and capacitor in parallel, from here referred to as an RC parallel circuit.

Unlike physical circuit elements, the resistance and capacitance magnitudes may vary over the experimental potential range applied to the device. Moreover, the circuit used to represent the device may vary with the applied potential, as physical processes in the device are often limited to a defined potential range. For example, before photocurrent onset, a large, low-frequency (10 kHz to 1 Hz) resistance has been previously observed in EIS studies of buried photocathode devices.^3,4^ This resistance corresponds to the differential resistance of the *JV* curve in this potential region and has been previously termed the charge-transfer resistance. It is attributed to a barrier at the a-TiO_2_|electrolyte interface, which was observed using the dual-working electrode (DWE) technique on a similar buried junction photocathode.^12^ As the onset potential is approached, this barrier decreases until the Fermi energy of the a-TiO_2_ is in line with the H^+^/H_2_ electrochemical potential. Correspondingly, the magnitude of the observed charge-transfer resistance becomes negligible as the photocurrent onset is reached.

Ambiguity in EIS analysis can arise when there are two or more distinct semicircles in the Nyquist plot occurring at the same potential. In this case, there are multiple ECs that can be used to model the data.^13^ With such ambiguity, choosing the correct EC for these systems goes hand in hand with understanding the physical mechanisms occurring within the device. Previously, it was reported that a-TiO_2_-protected photocathodes, specifically p-Si, Sb_2_Se_3_, and Cu_2_O, exhibit this ambiguity at potentials negative of photocurrent onset and continuing into the current plateau region.^4^ Two processes have also been previously reported during hydrogen evolution conditions for unprotected p-Si and p-InP photocathodes; however, data in the current plateau region was not obtained.^14−16^ Furthermore, based on the trends of the two processes with respect to potential, it is likely that only one process remains in the current plateau region for these unprotected devices. For the a-TiO_2_-protected devices, one of the two processes that persist in the current plateau region can be attributed to the semiconductor through the depletion layer capacitance, regardless of the EC used.^4^ This allowed important insights into device operation, although obtaining certainty regarding the correct EC was outside the scope of that report. Nevertheless, it remains important to resolve this ambiguity due to the extent that semiconductor|a-TiO_2_ heterojunctions are used in PEC literature.^17−21^

Amorphous TiO_2_ itself has many interesting properties that are behind its popularity as a protective layer. It has high chemical stability over a wide range of pH values, is a transparent window with respect to a large portion of the solar spectrum, and also serves as an antireflection layer.^1,2,22^ As it is deposited using atomic layer deposition (ALD), it forms conformal layers over structured semiconductors as well, which is useful for semiconductors that benefit from nanostructuring due to short minority carrier diffusion lengths.^23,24^ It has special significance for photoanodes, because a wide defect band (fwhm = 0.88 eV) facilitates hole transfer to the solution, which would otherwise be blocked by the position of the valence band.^2,25−27^ However, it is worth noting that when a-TiO_2_ is used to protect photocathodes, this defect band is not expected to play a role during device operation because it is likely that photogenerated charges pass through the conduction band of a-TiO_2_ to perform the HER.^25^ Note that, due to the amorphous nature of the a-TiO_2_, conduction may involve a hopping process. However, because a-TiO_2_ is very thin (100 nm), it is not expected to impede the device performance.

In this report, we resolve the EC ambiguity of a-TiO_2_-protected photocathodes by using a model p-Si system. Investigation of the effect of different overlayers on both device performance and corresponding impedance response reveals that the ambiguity arises from the presence of a-TiO_2_ itself. Furthermore, using the well-defined properties of crystalline silicon, we show that of the three possible ECs with which to model these devices, the Maxwell circuit is the only one to show consistency between devices with and without the a-TiO_2_ protection layer. Therefore, in addition to electron transfer during hydrogen evolution, a parallel charging process also occurs in these devices. This charging process is investigated by first removing intercalating species from the electrolyte solution, and also by interrupting communication between the p-Si substrate and a-TiO_2_ protection layer.

## Results and Discussion

### Voight vs Maxwell ECs for p-Si|a-TiO_2_|Pt Photocathodes

**Figure 2:**
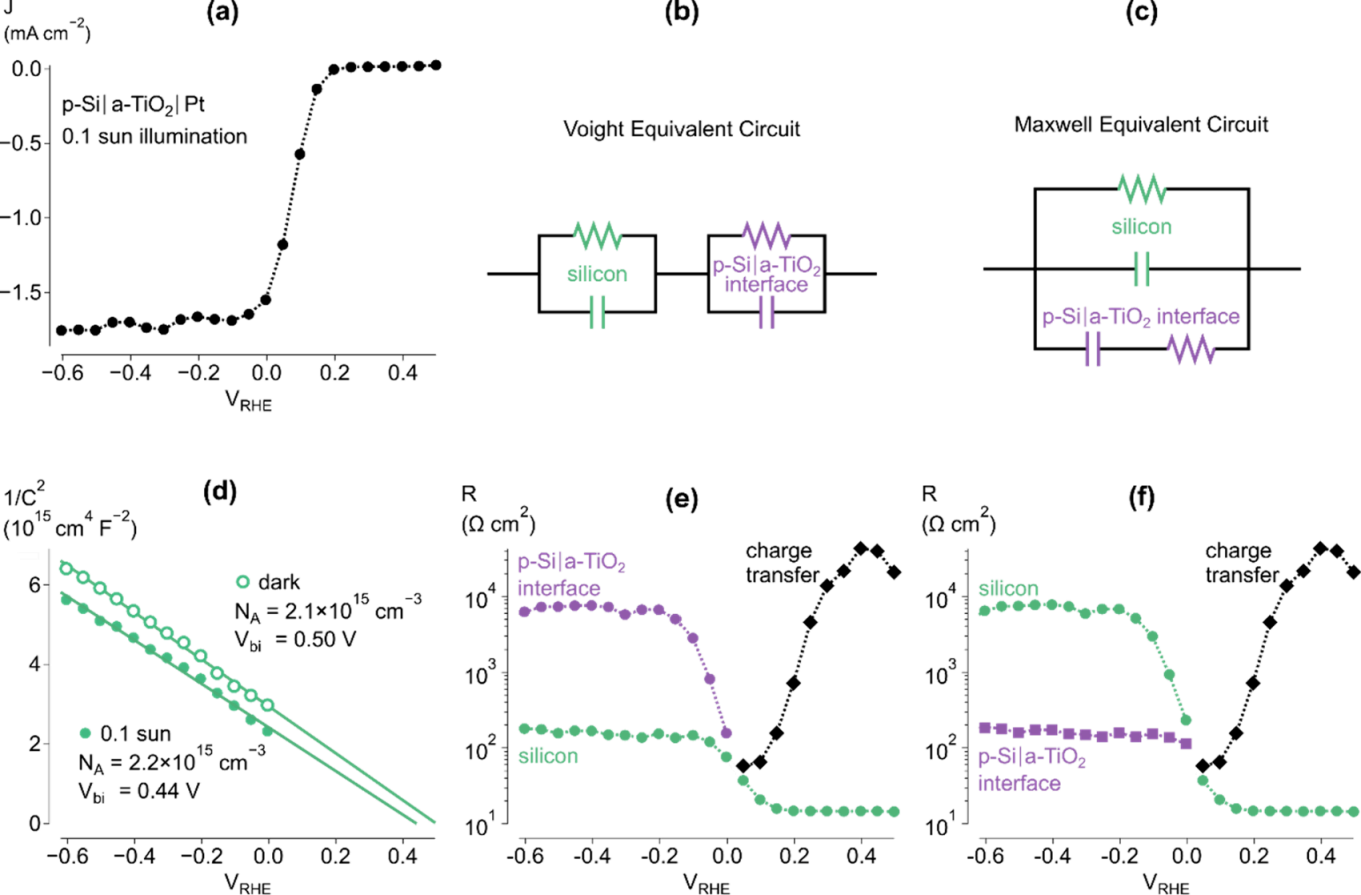
(a) Steady-state current–voltage curve of a p-Si|a-TiO_2_|Pt device under 0.1 sun illumination in 1 M H_2_SO_4_. (b) Voight and (c) Maxwell ECs used to fit the corresponding EIS data at voltages negative of 0 V vs RHE. (d) Mott–Schottky plot of the high-frequency capacitance under 0.1 sun illumination and in dark conditions, as labeled. The extracted doping densities correspond to p-Si, where the slight increase in doping under illumination can be attributed to photogenerated charges. The built-in voltage (*V*_bi_) represents the voltage of the p-Si|a-TiO_2_ junction. 0.1 sun data are taken from Maxwell conversion. Resistances obtained using the (e) Voight and (f) Maxwell circuits, showing significant differences in the obtained results at potentials negative of 0 V. For voltages positive of 0 V vs RHE, a Voight circuit was used, as determined previously.^4^ The surface charge-transfer resistance is shown in black. Here, the charge-transfer resistance corresponds to the differential *JV* curve of the buried junction device.

To understand the consequences of choosing one EC over the other, the impedance response of a p-Si|a-TiO_2_|Pt photocathode device was first revisited. Figure 2a shows the current–potential response of a typical device under 0.1 sun illumination. This light intensity was used to reduce noise originating from stochastic H_2_ bubble formation and detachment on the electrode surface. The a-TiO_2_ deposition on the p-Si used the same procedure as previously reported, which resulted in conformal films observed via SEM.^12^ Cross-sectional SEM was also performed here to confirm the presence of Ti and O in the deposited layer, as shown in Figure S1. The thickness of the film was confirmed via ellipsometry. The deposition of Pt using a pulsed PEC method was confirmed with XPS and AFM, as shown in Figure S2.

Processes at potentials positive of photocurrent onset have been described previously and can be modeled using the Voight circuit.^4^ Briefly, in Figure 2e,f, the charge-transfer resistance is a low-frequency process (10 kHz to 1 Hz) and reflects the differential resistance of the *JV* curve in this region. It corresponds to a thermodynamic barrier at the a-TiO_2_|electrolyte interface, which was observed in a similar buried junction photocathode device using a DWE technique.^12^ As the potential is moved from 0.5 V toward the photocurrent onset, the height of this barrier is reduced. Importantly, the band bending of the p-Si in this potential region, which is in slight depletion, remains constant. Therefore, an ac perturbation affects only the a-TiO_2_|electrolyte barrier. Although recombination of photogenerated charges is not due to unfavorable band bending in the p-Si, the recombination current is still observed as a high-frequency process (500 to 10 kHz). This is shown in green to indicate that the charges are recombining in the p-Si. The corresponding capacitance of the charge-transfer resistance is the depletion capacitance of the a-TiO_2_, as shown through Mott–Schottky analysis in Figure S3.

Prior to photocurrent onset, it is possible that contributions from multiple other interfaces influence the Nyquist plot of a PEC device. The TiO_2_|Pt, TiO_2_|electrolyte, and Pt|electrolyte interfaces have resistances associated with charge transfer across them; however, EIS is not able to deconvolute them, as only one process (semicircle) is observed. The Pt|electrolyte interface specifically is not observed as a separate process here due to the very low overpotential of Pt for the HER. For catalysts with a slightly higher overpotential for the HER or that require an activation step, such as RuO_*x*_, a separate process is observed prior to onset.^4^

At potentials negative of the photocurrent onset, and continuing into the current plateau region, two distinct semicircles are observed in the Nyquist plot and there is currently ambiguity regarding the choice of EC. Each of these semicircles represents a process with an associated resistance and capacitance occurring within the system. Parameters extracted from the high- and low-frequency semicircles have been labeled as silicon and the p-Si|a-TiO_2_ interface, respectively. The reasons behind these designations will be clarified throughout this report. Two EC options, the Voight and Maxwell circuits, are shown in parts b and c of Figure 2, respectively. Figure S4 shows that the quality of the fit to the raw Nyquist data is the same regardless of whether the Voight or Maxwell circuit is used to model the data. Moreover, the time constants of the system are independent of the EC used and are shown in Figure S5 for reference.^28^ The third option is the nested circuit; however, as shown in Figure S6, the Voight and nested circuits result in indistinguishable fits. Moving forward, the Voight circuit will be used to represent both the Voight and nested ECs and be compared against results using the Maxwell circuit.

Assigning one of these ECs over the other changes the interpretation of charge flow through the device. In the Voight and nested circuits, charges must pass through both resistors, which could imply a rate-limiting step for an overall process to occur if both time constants τ (obtained through τ = *RC*) represent a rate of charge transfer through a component of the device. It is worth noting that the nested EC has been used frequently to describe surface trap-mediated charge transfer from photoanodes such as Fe_2_O_3_, as well as limiting interfaces in halide perovskite solar cells.^29,30^ On the other hand, in the Maxwell EC, conduction through the circuit occurs through only one of the resistors in the low-frequency limit (the Si resistance in Figure 2c).^31^ This resistance represents the differential resistance of the device in operation taken from the *JV* curve and is the result of all of the photophysical and electrochemical processes taking place within the system.^31^ The additional branch of the Maxwell EC represents a short-range relaxation process, for example charge storage, occurring in parallel to conduction through the device.^13^

There are two ways to obtain the extracted parameters from both the Voight and Maxwell circuits. The Nyquist plots can be fit to both circuits separately, or transformation equations can be used to convert between ECs based on a single fit. The second strategy has been used here to convert parameters from a Voight circuit fit to those of a Maxwell circuit through the equations
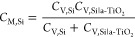
1
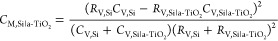
2
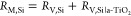
3
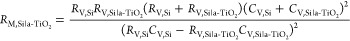
4where M and V subscripts indicate the Maxwell and Voight circuits, respectively.^13^ Additional subscripts correspond to circuit elements in Figure 2b,c.

It is important to note that the p-Si|a-TiO_2_ interface capacitance has been obtained using a constant-phase element (CPE), as opposed to a capacitor, as experimentally it is observed as a slightly flattened semicircle. The presence of a CPE is attributed to a distribution of capacitance values with respect to perturbation frequency arising from, for example, a variance of the double-layer capacitance on a rough electrode. As such, the corresponding RC time constant of double-layer charging across a rough electrode can vary.^32^ As opposed to a capacitor, which only conserves the charge, the CPE has a resistive component and therefore dissipates some of the charge. The parameter *Q* is the value of the CPE, while ϕ is an exponent describing the extent of deviation from the ideal capacitive behavior. The value of ϕ is constrained between 0.8 and 1.0 during fitting. A CPE with an exponent below 0.8 does not reasonably represent a capacitor, whereas a CPE with an exponent of 1.0 is an ideal capacitor. The pure capacitance is estimated using *C* = *Q*^1/ϕ^*R*^1/ϕ–1^, where *R* is the corresponding resistance.^32^ It is with the estimated pure capacitances that the transformation equations are used to convert between the Voight and Maxwell circuits. Figure S7 shows that using the transformation equations in this way results in p-Si|a-TiO_2_ interface capacitance values that differ slightly from those obtained when the data are fit directly to the Maxwell circuit. However, this discrepancy does not affect the analysis or conclusions of this work.

The Si and p-Si|a-TiO_2_ interface capacitances do not vary significantly when transforming from the Voight to Maxwell EC. This is because, again shown in Figure S6, the capacitances obtained using the Voight circuit are separated by approximately 3 orders of magnitude. Therefore, the eqs 1 and 2 collapse to *C*_M,Si_ = *C*_V,Si_ and *C*_M,interface_ = *C*_V,interface_. For this reason, the flat-band potential and doping density of Si can be extracted using Mott–Schottky analysis regardless of the EC used. This is why the EC did not need to be resolved in previous reports because it did not interfere with extracting these key parameters regarding the emerging semiconductors being studied.^4^ The capacitance, *C*, is used in Mott–Schottky analysis through the equation
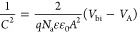
5where *q* is the electronic charge, *N*_a_ is the acceptor doping density, ε is the dielectric constant of silicon, ε_0_ is the permittivity of free space, *A* is the active area of the device, *V*_bi_ is the built-in voltage of the junction, and *V*_A_ is the applied potential. Note that this is slightly different from a conventional Mott–Schottky analysis of a semiconductor|electrolyte contact, where the built-in voltage would also represent the flat-band potential of the semiconductor. In the buried junction used in this study, the flat-band potential is not determined because, at potentials positive of the onset, a change in the applied bias affects only the energetics of the a-TiO_2_|electrolyte interface and not the band bending of p-Si.

In this report, Mott–Schottky analysis is used to confirm the known doping density of the Si semiconductor and, importantly, attribute the corresponding resistance to processes within the Si. The resistivity of the Si wafers used to fabricate the PEC devices is between 1 and 30 Ω cm, corresponding to a doping density between 4.5 × 10^14^ and 1.5 × 10^16^ cm^–3^. Figure 2d shows the Mott–Schottky plots of the high-frequency capacitance in both dark and 0.1 sun conditions, which give doping densities of 2.1 × 10^15^ and 2.4 × 10^15^ cm^–3^, respectively. The doping density and flat-band potential can be attributed solely to the Si here because Si|a-TiO_2_ is an asymmetric pn junction with Si having a much lower doping density than a-TiO_2_. Therefore, we can be confident that the capacitance and corresponding resistance in parallel can be attributed to the Si layer in this PEC device.

Unlike the capacitance values, the extracted resistances provide a way to distinguish between the Voight and Maxwell circuits. The Si and p-Si|a-TiO_2_ interface resistances are shown in Figure 2e,f for the Voight and Maxwell fits, respectively. The magnitudes of the Si and p-Si|a-TiO_2_ interface resistances differ by almost 2 orders of magnitude when fit with the Voight EC and effectively invert with respect to each other when transforming to the Maxwell EC. Again, this is due to the transformation in eqs 3 and 4 simplifying for *R*_V,Si_ ≪ *R*_V,p-Si|a-TiO_2__. Note that when fitting with the Maxwell circuit, the diameter of the semicircle no longer corresponds to the resistance of that element, as is the case for the Voight circuit.

With the goal of assigning either the Voight or Maxwell circuit to physical processes occurring in the p-Si|a-TiO_2_|Pt device, a-TiO_2_ was replaced with other protection layers to obtain complementary data. By continuing to use p-Si as the semiconductor, the ability to assign the Si capacitance and resistance of the device during operation was maintained. Furthermore, in the previous study where the EC ambiguity was observed, the common factor between device types was the a-TiO_2_ itself. It is likely that the ambiguity arises due to one of the properties of this protection layer.

### Altering the Protective Overlayer

Two alternate protection layers were investigated. First, the Si native oxide (SiO_*x*_) was used because it is stable in the acidic solutions used here for the HER. Second, the a-TiO_2_ was crystallized at 500 °C, resulting in an anatase protection layer. In addition to changing the phase of the protection layer, this also removed the wide defect band in the TiO_2_ bandgap.^2^ The phase change and defect band removal are shown with XRD and XPS, respectively, in Figure S8.

**Figure 3:**
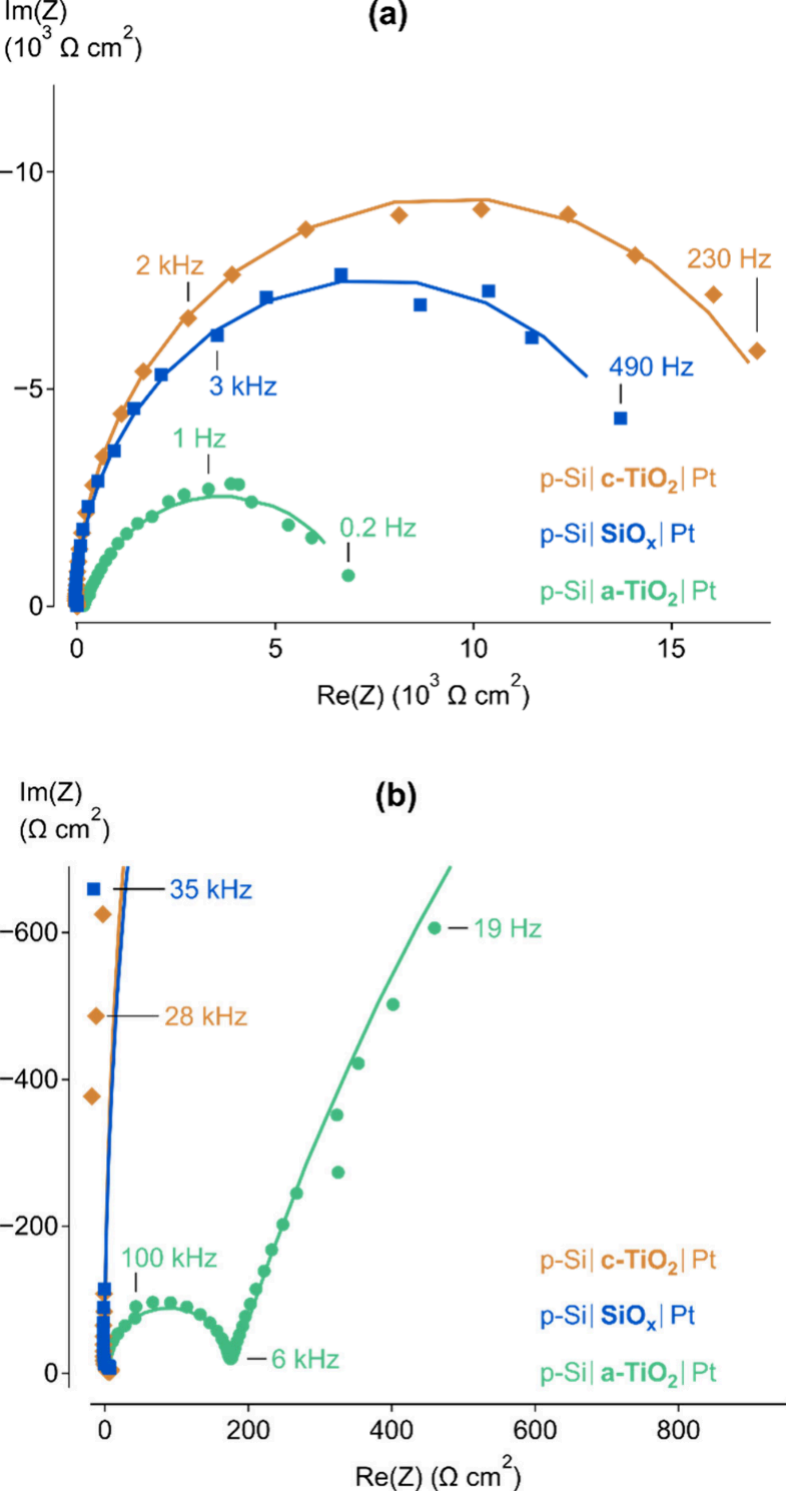
(a) Nyquist plots obtained under 0.1 sun illumination at −0.55 V vs RHE for devices with different protection layers, as listed. (b) High-frequency region of the same Nyquist plots, showing the presence of an additional semicircle for the p-Si|a-TiO_2_|Pt device only. Select frequencies have been labeled for each device type.

Although the change in the protection layer has no discernible effect on the device performance (Figure S9), a significant difference is observed in the Nyquist plots negative of the onset potential. Figure 3 compares these Nyquist plots, which were obtained under 0.1 sun illumination at −0.55 V vs RHE. At this potential, all devices are operating in depletion and show a saturated photocurrent. Clearly, for the two alternate protection layers, only one semicircle is visible and therefore the RC parallel circuit can be used to extract resistances and capacitances for these devices during the HER. The change in the device response can also be observed using distribution of relaxation times analysis, which is shown in Figure S10. Therefore, the low-frequency process observed in a-TiO_2_-protected devices originates from the presence of a-TiO_2_ itself.

As expected, fitting the SiO_*x*_- and c-TiO_2_-protected devices to the RC parallel circuit shows that the observed semicircle can be attributed to processes associated with Si. This is because the doping densities obtained with Mott–Schottky analysis once again correspond to the known values (Figure S11). Just as the RC parallel circuit provides unambiguous values for the Si capacitance, there are now unambiguous values for the Si resistance of these devices during the HER.

### Resolving the EC Ambiguity

**Figure 4:**
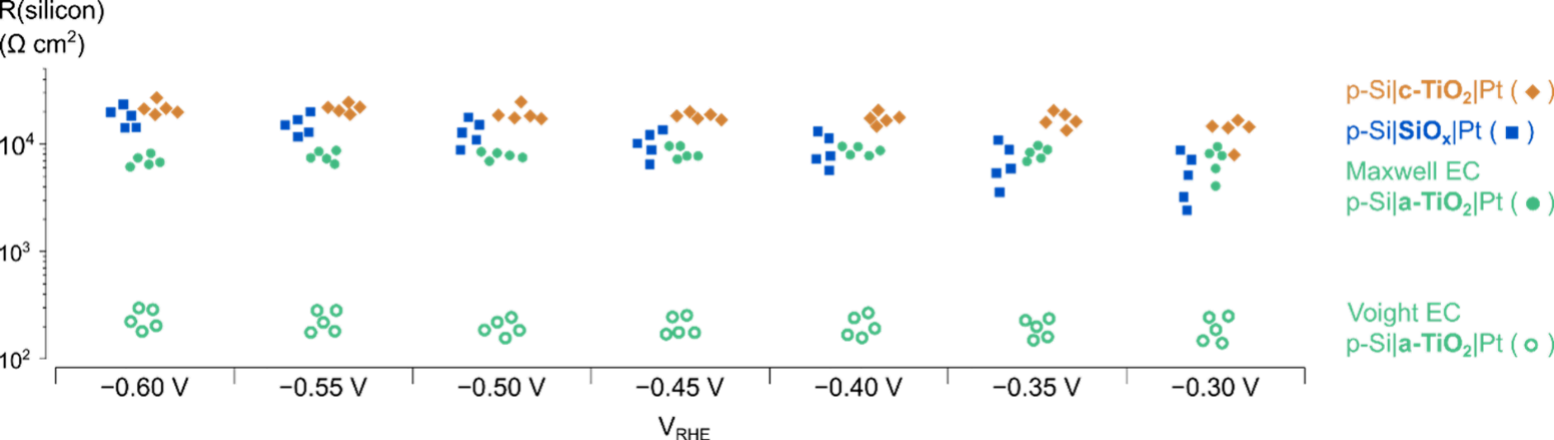
Si resistance values in parallel with the depletion layer capacitance, extracted from EC analysis of devices operating under 0.1 sun illumination. For devices protected with a-TiO_2_, two data sets are shown which correspond to each of the possible ECs, showing that the Maxwell circuit parameters closely match those of the other device types.

The Si resistances obtained for devices with c-TiO_2_ and SiO_*x*_ protection layers were compared to those from both the Voight and Maxwell fits for devices with a-TiO_2_ protection layers. Figure 4 shows these results for potentials from −0.30 to −0.60 V vs RHE, with five replicates per device type. For this potential range and illumination intensity, all devices have reached the saturation current regime. As shown, using the Maxwell circuit for a-TiO_2_-protected devices gives Si resistances consistent with the values obtained for SiO_*x*_- and c-TiO_2_-protected devices. Alternatively, using the Voight circuit for a-TiO_2_-protected devices results in values that are over 1 order of magnitude less. Although results have so far focused on devices under 0.1 sun illumination for ease of analysis, these conclusions are maintained for higher light intensities as shown in Figure S12.

At this point, there is an opportunity to resolve the EC ambiguity of the p-Si|a-TiO_2_|Pt device during the HER. If the resistance associated with Si is expected to be the same regardless of protection layer, given very similar device performances, then Figure 4 indicates that the Maxwell circuit is appropriate to describe device operation for the a-TiO_2_-protected devices. If not, the Voight circuit continues to be a valid option. Figure 4 does highlight that altering the protection layer between c-TiO_2_ and SiO_*x*_ does not change the resistance of Si significantly. However, as mentioned, a-TiO_2_ has unique properties such as a defect band and the ability to intercalate ions that may affect device operation. Therefore, it is important to have a clear understanding of the physical mechanism of the Si resistance during device operation before determining the appropriate EC.

The resistance of a pn junction in the dark (also called the shunt resistance) is well documented and will be used to first discuss the response of the PEC devices in the dark, before moving on to illuminated conditions. The slope of the dc current–voltage response defines the resistance, *R*, through *R* = d*V*/d*I*, where *V* and *I* are the voltage and current, respectively. This resistance, found through dc characteristics, is also defined to be the ac resistance, determined with EIS. In forward bias, majority carriers flow through the junction and there is a small resistance associated with this process. As the applied voltages move the device toward depletion, an increasing thermodynamic barrier for the majority carriers prevents current flow, corresponding to a high differential resistance. For ideal diodes, the resistance in this region is infinite, as the slope of the current–voltage response is zero. For nonideal diodes, a small leakage current of minority carriers exists in depletion. These minority carriers can arise from thermal promotion via trap states such as from crystal or interface defects (the inverse of SRH recombination).^33^ This mechanism of charge flow in depletion corresponds to a reduced resistance of the junction so that it is no longer infinite. Again, this can be observed with both dc and ac measurements.

The same principles apply to the asymmetric pn+ junction in the PEC devices here. The junction (Si) resistance increases as the devices goes further into depletion. Figure S13 shows the agreement of Si resistances found using both dc and ac methods in the dark for an example device with each protection layer. Also shown are band diagrams comparing the Si band bending at potentials before and after onset. The change in band bending affects the thermodynamic barrier seen by the majority carriers and ultimately the resistance of the junction. Note that for the PEC devices, although the junction resistance is small at more positive potentials, a net current is not observed due to the lack of a sufficient concentration of acceptors at an appropriate energy level in the electrolyte solution.

**Figure 5:**
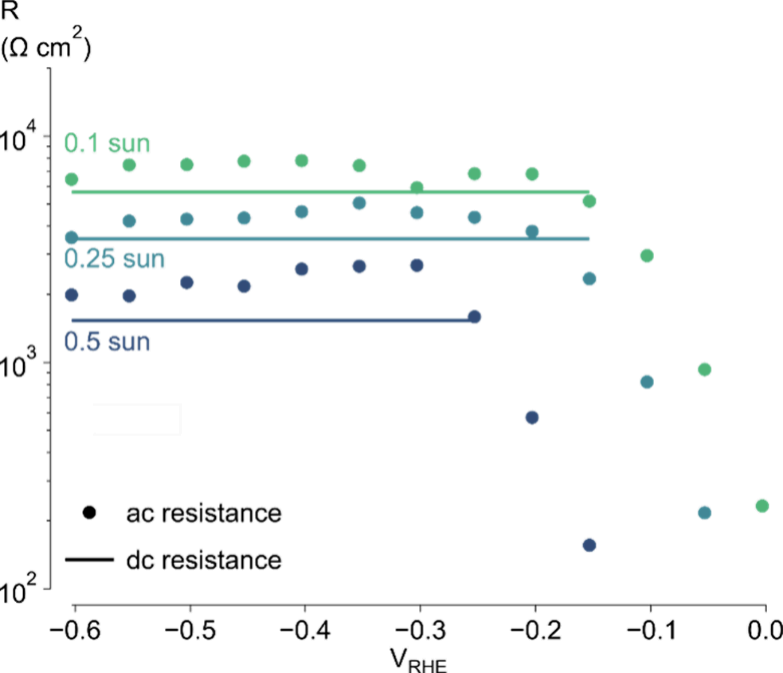
Markers showing ac Si resistances obtained with EIS and the Maxwell circuit. Lines show device resistances taken from the inverse slope of the current–voltage response in the potential region where the current is approximately constant. A linear fit over this potential range was used, due to the influence of bubble formation and release on the sign of the local slope.

Under illumination, excess minority carriers are generated which can pass through the junction. With this additional mechanism of current flow through the junction, the corresponding resistance at a defined potential is decreased. However, the resistance of the junction continues to reflect the slope of the current–voltage curve. This has been shown for pn^+^ photovoltaic devices under various illumination intensities, where an increase in the illumination intensity corresponds to a decrease in the dc and ac device resistance at a defined reverse-bias voltage.^34^ Again, this is also shown for the PEC devices in this study. Importantly, for devices protected with a-TiO_2_, the dc resistance agrees with the ac Si resistance when the Maxwell circuit is used to fit the impedance data, as shown in Figure 5. Here, taking the slope of the current–potential curve is not straightforward over the entire voltage range of interest. Due to the random current fluctuations during the HER, the local slope around a given bias voltage can be negative. However, these negative values do not represent device operation as the current fluctuations themselves are due to random bubble formation and detachment on the surface of the device. Therefore, a linear fit of the data has been taken in regions where the current is approximately linear with a change in potential, and subsequently used to represent the dc resistance.

Device resistances on the order of 10^4^ Ω cm^2^ are low compared to high-performing devices with a Si photoabsorber. For example, a Si photovoltaic under 0.2 sun illumination has a corresponding resistance of approximately 10^5^ Ω cm^2^.^34^ Therefore, the lower resistances of the devices in this study could indicate a leakage current due to direct contact between the back of the p-Si and the electrochemical interface. However, the high performance Si photovoltaic achieves a corresponding current density of approximately twice that of the devices in this study, when differences in incident light intensity are accounted for. Therefore, it is likely that these resistance values arise instead from the loss of photogenerated charges, as already seen in the difference between the built-in voltage of the junction (0.5 V as determined by Mott–Schottky; Figure 2d) and the photocurrent onset (0.2 V vs RHE).

Having established the origin and behavior of the Si resistance under illumination, the appropriate EC can be determined to describe p-Si|a-TiO_2_|Pt devices operating in reverse bias. First of all, despite having different protection layers, which has significant effects on the observed Nyquist plots, all devices have very similar onset voltages, fill factors (FFs), and saturation currents, as shown in Figure S9. With similar current through all junctions at a defined voltage, it is expected that the resistances of all junctions will be similar. Referring back to Figure 4, this is only achieved when using the Maxwell circuit to model the device during EIS. Furthermore, the expected equality between the Si ac resistance and the dc resistance, regardless of illumination intensity, is only observed when using the Maxwell circuit (Figure 5). Large deviations from this established behavior are observed if the Voight circuit is used to model the small signal response of the device, as the contribution of the junction (Si) to the overall resistance will be 2 orders of magnitude smaller for a-TiO_2_-protected devices, despite the fact that the behavior of the junction is not expected to differ between device types at these potentials. Therefore, the data here confirm that the Maxwell circuit best describes the underlying operation of p-Si|a-TiO_2_|Pt devices performing the HER.

### Physical Mechanism of the Nonfaradaic Process

As the additional process in the Maxwell circuit is a nonfaradaic charging process, two possible physical mechanisms were investigated experimentally. The first mechanism investigated was intercalation of ions into the a-TiO_2_ structure. Although known to occur in both c-TiO_2_ and a-TiO_2_, proton intercalation occurs over a wider voltage range for a-TiO_2_. Therefore, it has the potential to be detected with EIS at voltages negative of the HER onset. Furthermore, the 1 M H_2_SO_4_ electrolyte provides an ample concentration of protons at the a-TiO_2_ surface to be intercalated. Second, a charging process at the p-Si|a-TiO_2_ interface was investigated by insertion of a metallic interlayer, which effectively screened interactions between the two materials.

Testing whether the nonfaradaic process is due to intercalated protons in the a-TiO_2_ material was carried out by performing EIS in an aprotic solvent during ferrocenium reduction. To remove all possible intercalating species from the electrolyte, tetrabutylammonium hexafluorophosphate was used as a supporting electrolyte in acetonitrile. Subsequently, a lithium hexafluorophosphate electrolyte was used for comparison purposes. As with the proton, Li^+^ is also able to intercalate into a-TiO_2_. Therefore, if intercalation is the root cause of the nonfaradaic process observed in a-TiO_2_-protected photocathodes, a clear difference between experiments using these two different supporting electrolytes should be observed.

Ultimately, this clear difference was not observed as the Nyquist plots for devices operating in aprotic solvents continued to show two simultaneous processes during the PEC reduction. This is shown in Figure S14, where again the resistances of two processes are observed to increase at voltages negative of the onset voltage. The magnitudes of these two resistances are approximately in agreement with those of a device operating in a protic electrolyte at the same current density. Furthermore, no change in the capacitance associated with a-TiO_2_ was observed in the aprotic tests involving different supporting electrolytes (Figure S14d). That is, introduction of an intercalating species did not change the magnitude of the nonfaradaic capacitance. This indicates that the process of intercalation during the PEC reduction is not the origin of the additional process in a-TiO_2_-protected photocathodes during the HER.

Screening charges between the p-Si and a-TiO_2_ through insertion of a metallic interlayer required two energetic considerations. First, the metallic contact to p-Si had to be rectifying, and second, the metallic contact to a-TiO_2_ had to be ohmic. This was achieved using a metallic Ti layer, which was sputtered on the p-Si substrate before deposition of the a-TiO_2_ through ALD. After subsequent deposition of a Pt catalyst, the devices could be operated as a PEC for the HER. Significant light attenuation was introduced into the device due to this metallic interlayer, which was overcome by increasing the incident illumination to the equivalent of 1.5 suns at the front of the electrochemical cell to reach similar photocurrents as with the standard devices.

**Figure 6:**
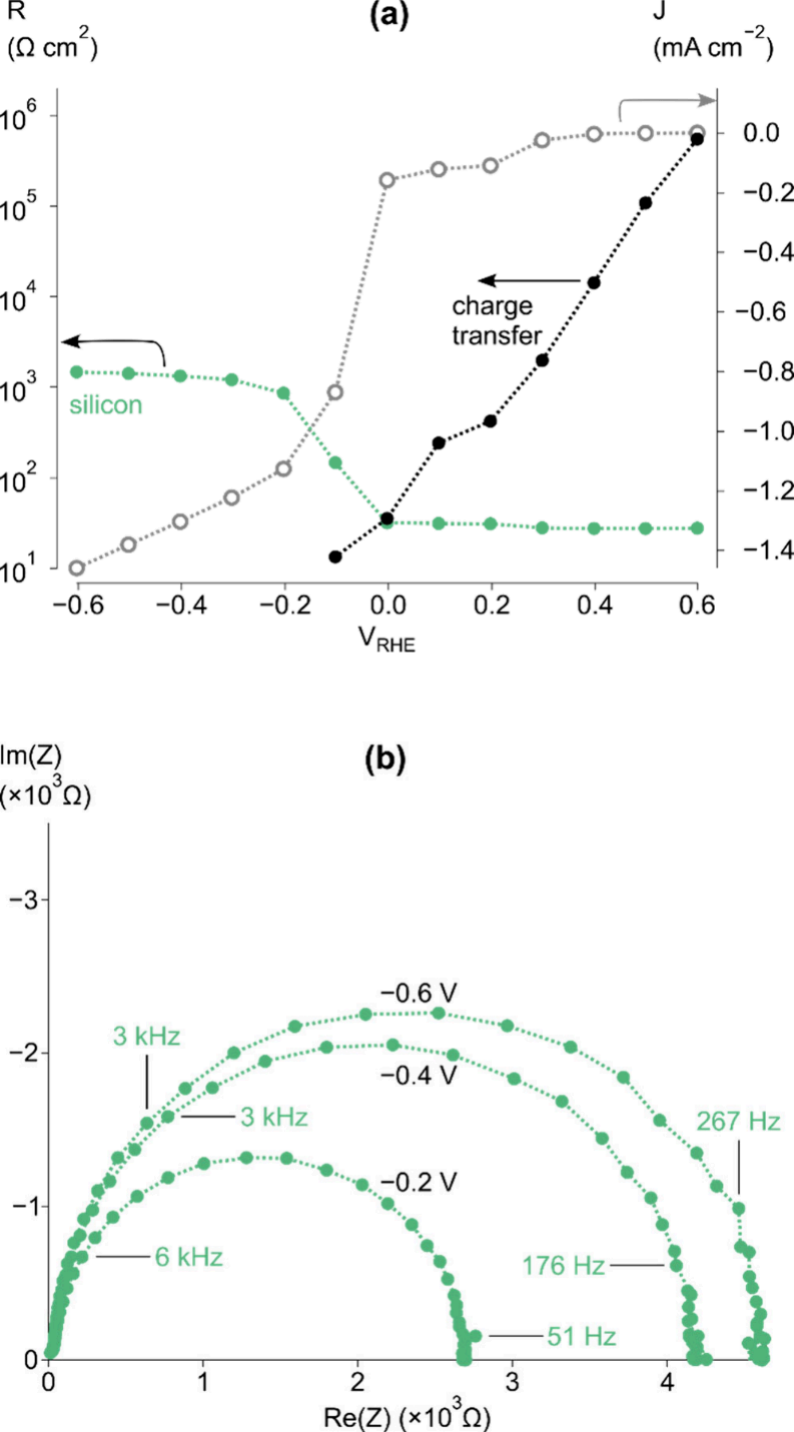
(a) Current–voltage response and resistances of a p-Si|Ti|a-TiO_2_|Pt device under 1.5 sun illumination. (b) Nyquist plots at voltages vs RHE during the HER, as indicated. This emphasizes that there is only one process visible with EIS now that a metallic interlayer has been inserted between the p-Si and a-TiO_2_. Select frequencies have been labeled for each applied voltage.

The EIS results of PEC devices with a metallic Ti interlayer are shown in Figure 6. As with the standard p-Si|a-TiO_2_|Pt devices, a charge-transfer resistance before photocurrent onset is observed. At voltages negative of the onset voltage, this resistance becomes negligible and the silicon resistance increases. Importantly, only one process is observed in the Nyquist plots at voltages negative of the HER onset, showing a clear distinction between devices without the metallic interlayer. These Nyquist plots are shown in Figure 6b, where there is no indication of a second process occurring simultaneously with this silicon RC element.

As insertion of the metallic interlayer removed the observation of the nonfaradaic process, this indicates that the process itself originates at the interface between the p-Si and a-TiO_2_. Additional characterization methods would be required to resolve the exact mechanism; however, it remains likely that the defect band of the a-TiO_2_ plays a role. The defect band is not required in these devices to carry out the electrochemical reaction; however, it is still an electrochemically active band of states that has been shown to transport charges across interfaces. Furthermore, alignment of the silicon valence band with this defect band may also facilitate photogenerated charge transfer between these two materials.

## Conclusion

Due to the presence of two simultaneous processes observed with EIS during the HER, the EC with which to model p-Si|a-TiO_2_|Pt devices was previously ambiguous. This ambiguity had implications on the mechanism of charge transfer through these devices, and therefore was important to resolve. This was achieved by substitution of the a-TiO_2_ protection layer with both SiO_*x*_ and c-TiO_2_, which resulted in unambiguous resistance and capacitance values for the silicon substrate during the HER. Taking the silicon resistance found using EIS to be equivalent to the dc resistance, it was confirmed that the Maxwell circuit is the appropriate EC with which to model a-TiO_2_-protected devices during the HER.

The Maxwell circuit implies that there is a long-range, faradaic process in parallel with a nonfaradaic process. As the origin of the nonfaradaic process was determined to be associated with the a-TiO_2_ protection layer, the mechanism of this process was investigated further. Experiments in aprotic solution showed that proton intercalation into the a-TiO_2_ material is not the source of the nonfaradaic process, despite previous knowledge that this can occur in a-TiO_2_ in the potential range of interest here. However, insertion of a metallic interlayer, to screen the p-Si and a-TiO_2_ materials from each other, resulted in complete removal of the nonfaradaic process, as determined by EIS. This implies a charging mechanism exists at the interface between the p-Si and a-TiO_2_, which could involve the presence of the defect band states, despite the fact that these states do not influence the overall performance of these photocathode devices.

## Experimental Section

P-doped ⟨100⟩ Si wafers with a thickness of 525 ± 25 μm and a resistivity of 1–30 Ω cm were purchased from Si-Mat Silicon Materials. The wafers were sonicated for 10 min each in acetone, water with Deconex 11 UNIVERSAL, water, and isopropyl alcohol. They were then cleaned at 80 °C for 10 min in a 1:1:5 H_2_O_2_ (30%, Supelco Analytical)/NH_4_OH (25%, Honeywell)/water solution, followed by 10 min in a 1:1:5 H_2_O_2_ (30%, Supelco Analytical)/HCl (37%, Merck)/water solution. Then, the native oxide was etched through submersion in a 2% HF solution for 30 s. For devices with a SiO_*x*_ protection layer, this final etching step was not performed.

A PICOSUN R-200 ALD system was used to deposit 100 nm of a-TiO_2_ on the Si substrates using water and tetrakis(dimethylamino)titanium (TDMAT) as the O and Ti precursors, respectively. The sample stage was held at 120 °C during the deposition. A 1.6 s pulse of TDMAT and 7.0 s N_2_ purge was followed by a 0.1 s water pulse, followed by a 6.0 s N_2_ purge to deposit a single layer of a-TiO_2_. These steps were repeated until the desired thickness was achieved. The thickness of the a-TiO_2_ layer was confirmed using a J. A. Woollam Co. alpha-SE ellipsometer. Ligand incorporation in the a-TiO_2_ films is expected during the deposition and has been previously studied.^35,36^ For devices with a c-TiO_2_ protection layer, the samples were subsequently heated to 500 °C for 1 h on a hot plate in air.

Contact to the back of the device was made with GaIn eutectic (Ga 75.5%/In 24.5%, Merck). Ag paste (Loctite EDAG 1415M) was then used to fix a piece of Cu foil to the back, which acted as the current collector. The active area of the device was defined with epoxy (Loctite EA9466 and EA9461).

The Pt catalyst was deposited using a pulsed photoelectrodeposition method with 10 mM H_2_PtCl_6_ (ACS reagent, ≥37.50% Pt basis, Merck) in 2 M HCl. Under 1 sun illumination, the voltage was stepped between OCV (for 5 s) and a negative bias (for 0.25 s) that reduced Pt onto the surface of the device. For devices with a-TiO_2_ and c-TiO_2_ protection layers, the bias voltage was −0.15 V vs Ag|AgCl. For devices with the SiO_*x*_ protection layer, the bias voltage was −0.55 V vs Ag|AgCl. After 10 mC cm^–2^ of charge had passed, cyclic voltammetry in 1 M H_2_SO_4_ under 0.5 sun was performed (25–50 cycles) to condition the catalyst and improve the FF.

For all protic experiments, 1 M H_2_SO_4_ was used as the electrolyte. The counter electrode was isolated from the device using a Nafion membrane. H_2_ was bubbled off of a second Pt wire at 2 mA on a second channel of the potentiostat throughout the measurements, to maintain an equilibrium of H_2_ and H^+^ at the active area of the device.

For aprotic experiments, anhydrous acetonitrile (99.8%, Merck) was used as the solvent. Lithium hexafluorophosphate (battery grade, ≥99.99%) was used as purchased, and tetrabutylammonium hexafluorophosphate (for electrochemical analysis, ≥99.0%) was dried under a dynamic vacuum at 80 °C for 2 h. All electrolytes used for the aprotic experiments were made in an N_2_-filled glovebox.

The Ti interlayer was deposited via sputtering (Safematic CCU-010 sputter coater) with a Ti target (99.98%, Baltic Präparation e.K.). The purge and background gas was Ar. The target was presputtered to remove oxides before opening the shutter for deposition. The deposition current was 100 mA, and deposition pressure was 8.0 × 10^–3^ mbar.

TiO_2_ samples for XPS analysis were prepared on degenerately doped n-Si substrates (Siltronix, ⟨111⟩, 0.4–0.6 Ω cm resistivity, 500–550 μm thickness). Wafers were cleaned and etched prior to TiO_2_ deposition, as previously described. For Pt analysis, a p-Si wafer as described for PEC devices was used as a substrate. A Physical Electronics (PHI) Quantum 2000 XPS spectrometer was used to obtain XPS spectra. Monochromatic Al Kα radiation was generated from an electron-beam operating at 15 kV and 32.3 W. To calibrate the energy scale, Au and Cu reference samples were used. A pressure of 1 × 10^–6^ was maintained in the sample chamber. The electron takeoff angle was 45°, and the pass energy was 23.50 eV.

SEM was performed using a Zeiss GeminiSEM 450 field-emission scanning electron microscope. Images were obtained using a secondary electron detector, and elemental analysis was obtained using an X-MAX80, AZTec Advanced, Oxford X-ray detector.

Grazing-incidence XRD was performed using a Rigaku Smart Lab X-ray diffractometer with an omega angle of 0.5°. The preparation of the samples was the same as described for XPS analysis.

The FF was calculated using the equation , where *V*_mp_ and *J*_mp_ are the voltage and current density at the maximum power point of the device. *V*_oc_ was taken as the onset potential of the device, and *J*_sc_ was taken at −0.8 V vs RHE for all devices.

All electrochemical tests were performed with a Biologic SP-300 potentiostat. Devices were illuminated using a white LED source from Luxeon Rebel (SP-12-W5, cool white). EIS was performed using a single sine wave and a perturbation voltage of 10 mV. With logarithmic spacing, 70 frequency points between 7.000 MHz and 0.200 Hz were probed at each bias potential. Bias potentials were applied in 50 mV steps. The voltage perturbation started 30 s after the application of each bias potential. Nyquist plots were fit with *ZView* software from Scribner.

## Supporting Information Available

Cross-sectional SEM of p-Si|a-TiO_2_, AFM and XPS of the Pt catalyst, a-TiO_2_ and Si Mott–Schottky analysis, comparison of the EC fits, lifetime analysis, XRD and XPS comparisons of a-TiO_2_ and c-TiO_2_, device performance metrics, device band diagrams, and EIS results in aprotic electrolyte (PDF)
