# Supplementary material for: Operation of Amorphous TiO2-Protected Photocathodes Described with the Maxwell Equivalent Circuit
Source: ACS Appl Mater Interfaces. 2024 Oct 22;16(44):60084–93. doi: 10.1021/acsami.4c07588 (PMC11551960; doi:10.1021/acsami.4c07588)
Supplement: Supplementary file 1 — am4c07588_si_001.pdf [file am4c07588_si_001.pdf]

Supporting Information

Operation of Amorphous TiO_2 Protected Photocathodes Described with the Maxwell Equivalent Circuit

Erin Service[a], Thomas Moehl[a]*, S. David Tilley[a]*

[a]Department of Chemistry, University of Zurich, Zurich 8057, Switzerland

*Email: thomas.moehl@chem.uzh.ch

*Email: david.tilley@chem.uzh.ch

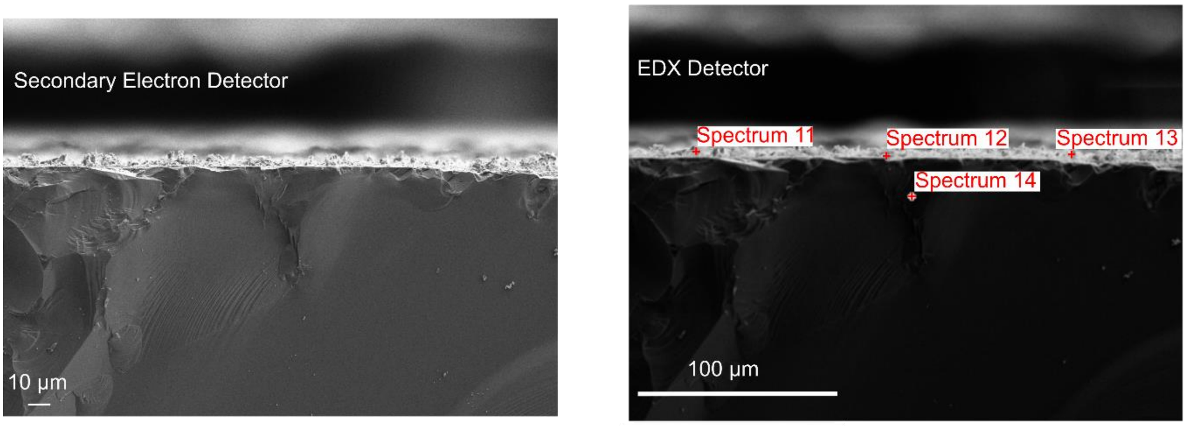

Figure S 1 Cross sectional SEM images of p-Si|a-TiO_2, using detectors as indicated. Elemental analysis was performed to confirm the presence of Ti and O, confirming deposition of a-TiO_2 on the Si substrate using ALD. Percentages of elements are shown in the table below, for each point scan indicated on the EDX image.

**Spectrum #**	**Si wt%**	**O wt%**	**Ti wt%**	**C wt%**
11	79.9	7.6	6.3	6.2
12	84.4	4.5	2.9	8.2
13	64.3	17.0	10.8	8.0
14	96.5	0	0	3.5

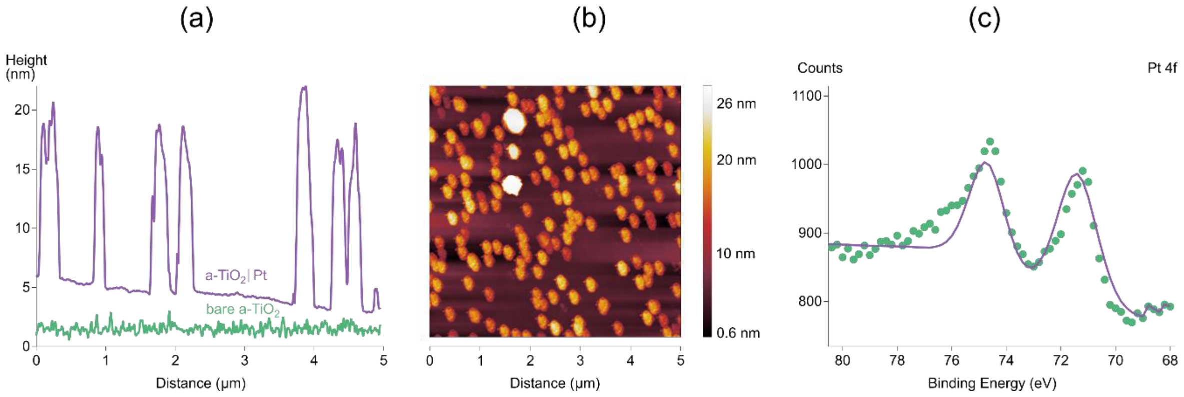

Figure S 2 (a) AFM height profile of both a bare a-TiO_2 layer and an a-TiO_2 layer with Pt deposited via pulsed photoelectrodeposition. (b) Map of AFM, showing distributed Pt particles on the surface of a-TiO_2. (c) XPS of a p-Si│a-TiO_2│Pt substrate, confirming the presence of metallic Pt on the surface of the electrode.

Deposition of the Pt catalyst on all devices was monitored through the number of coulombs passed (10 mC cm^{-2}), as different pulse voltages were employed for different overlayers, as outlined in the experimental section. Physically, the presence and morphology of Pt was confirmed using AFM and XPS, as shown above. The pulse voltage for these substrates was −0.05 V vs Ag│AgCl.

AFM shows that, in comparison to the flat layer of a-TiO_2 (green, Figure S2 (a)), the substrate with Pt has large pillars. These pillars are taken to be the Pt catalyst. In panel (b), the pillars are shown to be dispersed across the surface of the substrate in a fairly uniform manner. Panel (c) confirms the presence of Pt on the surface of the substrate, which indicates that the observed pillars are indeed the Pt catalyst.

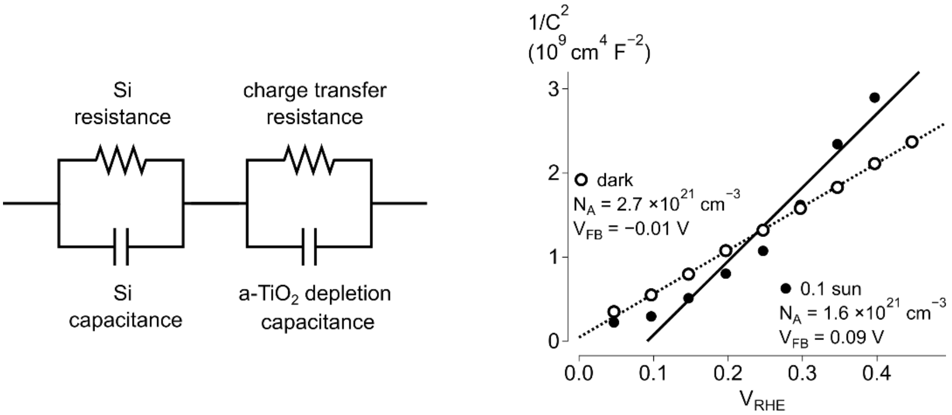

Figure S 3 Voight circuit used to fit the Nyquist plots of the p-Si|a-TiO_2|Pt photocathode before onset of photocurrent is shown of the left. On the right, Mott-Schottky plots of the a-TiO_2 depletion capacitance. Extracted flat-band values and doping densities agree with previous literature.

For an a-TiO_2 protected photocathode, it is possible to observe resistances originating from the Si, a-TiO_2 and the catalyst prior to onset, as previously described.[1] In the p-Si│a-TiO_2│Pt devices of this study, the catalyst resistance is not observed due to the high HER activity of Pt. Also, the Si resistance is small such that the charge transfer resistance at the a-TiO_2 surface dictates device performance in this potential region. The corresponding capacitance represents the depletion capacitance of the a-TiO_2, as Mott-Schottky plots have parameters corresponding to this layer. A dielectric constant of 33 was used to extract the doping density of a-TiO_2.[2] The extracted doping densities of over 1×10^{21} cm^{-3}, as labelled, would be extremely high for crystalline semiconductors. However, this material is known to have a high concentration of impurities due to incorporation of the ligands from the TDMAT precursor as well as a high concentration of vacancies.[3] The extracted flat-band voltages also agree with previous reports of amorphous TiO_2 films.[4,5]

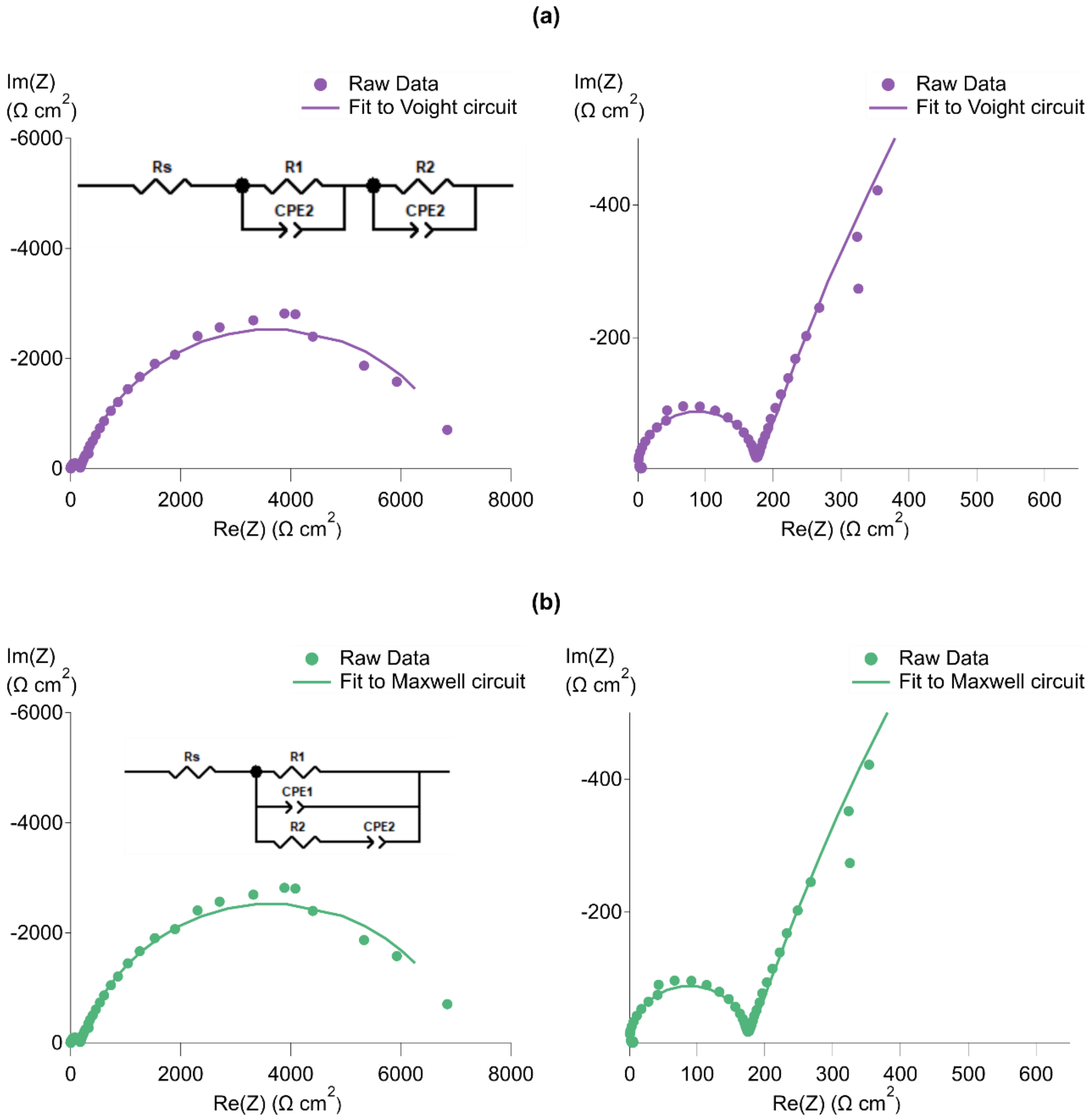

Figure S 4 (a) Nyquist plot fit to the Voight EC of an p-Si│a-TiO2│Pt device at −550 mV vs RHE. (b) the same Nyquist plot raw data, fit to the Maxwell EC. For both fits, the exponent of CPE1 was fixed to 1.0, rendering it a pure capacitor, and the exponent of CPE2 was fixed to 0.8. The Rs value was fixed to 1. The frequency range of collected data is from 7 MHz to 0.2 Hz.

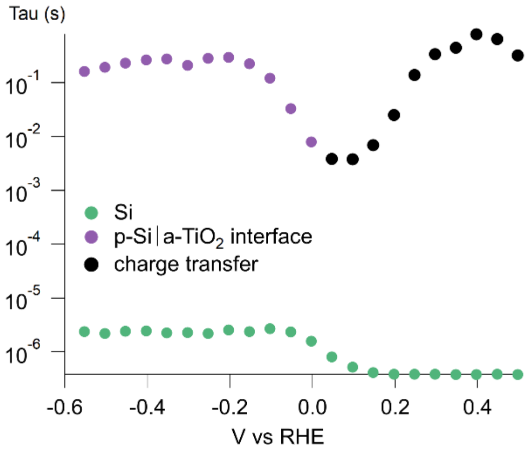

Figure S 5 Time constants obtained from the Nyquist plot fits to the Voight circuit (Tau = RC). Note that the time constants of the system are independent of the EC fitting.

(a)

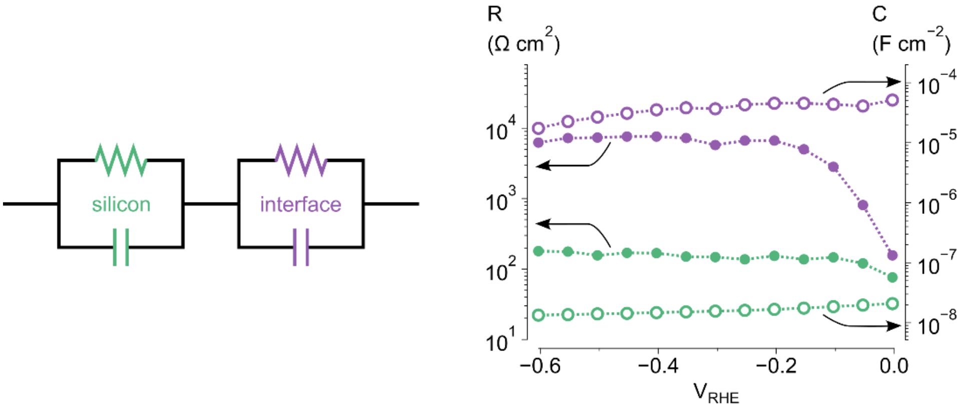

(b)

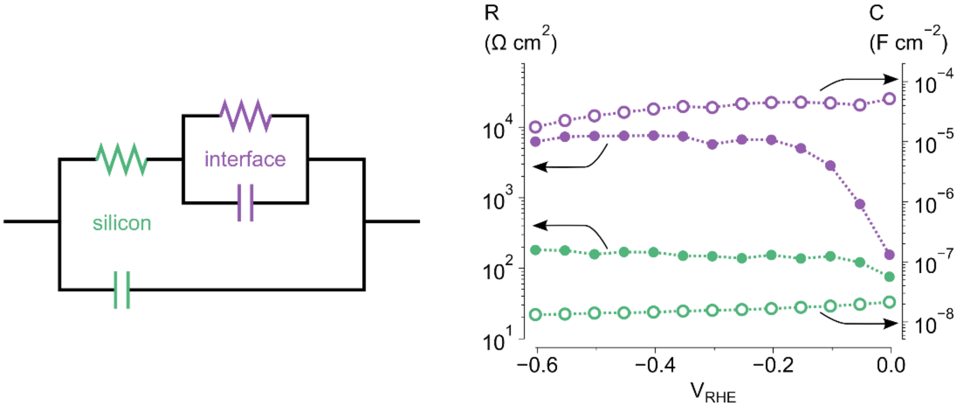

Figure S 6 (a) Voight circuit and corresponding resistances and capacitances obtained from that circuit. (b) Nested circuit and corresponding resistances and capacitances obtained from that circuit. For both, the silicon capacitance is identified using Mott-Schottky analysis, while the silicon resistance is the resistor in parallel to the silicon depletion capacitance.

Figure S6 shows parameters obtained after onset, when using both the Voight and nested (also called matryoshka or ladder type) equivalent circuit. Parameters from both these circuits were obtained by directly fitting the data to the respective equivalent circuit. Comparing the results, it is clear that the extracted parameters are effectively the same, and therefore the Voight and nested circuits are indistinguishable for p-Si|a-TiO_2|Pt devices under illumination and after the onset voltage. Nevertheless, the physical meaning of the two different ECs can imply different mechanisms.

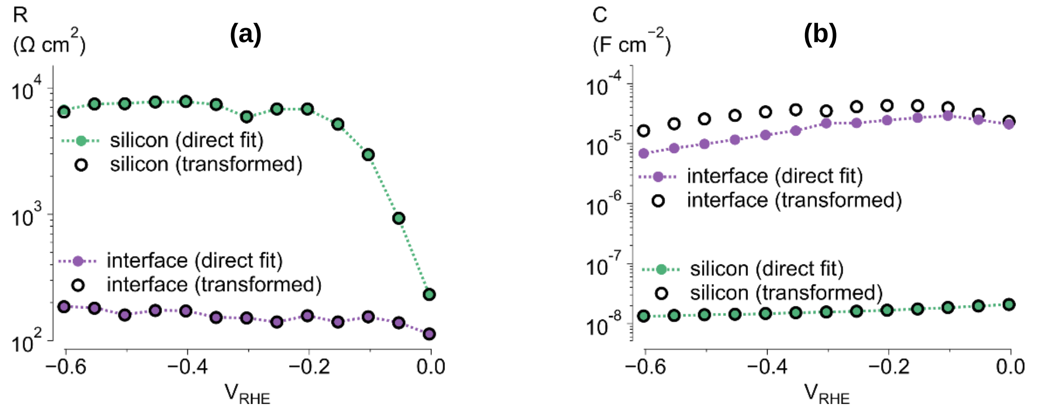

Figure S 7 (a) Resistances and (b) capacitances obtained when using the Maxwell circuit to model the illuminated p-Si|a-TiO_2|Pt device at voltages negative of the onset. Filled data points have been obtained when fitting the data directly to the Maxwell circuit, while data points represented with black circles have been obtained when converting Voight circuit parameters to Maxwell circuit parameters.

In Figure S7, the effect of using a capacitance obtained from a constant phase element (CPE) in the conversion equations from the Voight to Maxwell EC is shown. The values from both a direct fit and conversion are shown. For the converted values the raw Nyquist plots have first been fit using the Voight circuit, where a CPE was used for the interface capacitance. Then the CPE was converted to a capacitance using $C = Q^{1/\varphi}/R^{1/(\varphi-1)}$. This capacitance value was then used in the conversion equations.

Using the capacitances obtained from a CPE in the Voight EC and subsequently using the transformation equations has no effect on the values of the resistances obtained, when compared to fitting the data directly to the Maxwell circuit. The same can be said for the capacitance of silicon. However, there are discrepancies between the capacitance values obtained for the interface element. As the discrepancies are less than an order of magnitude, and these values are not used for any conclusions in the work, the conversion equations have continued to be used throughout this report.

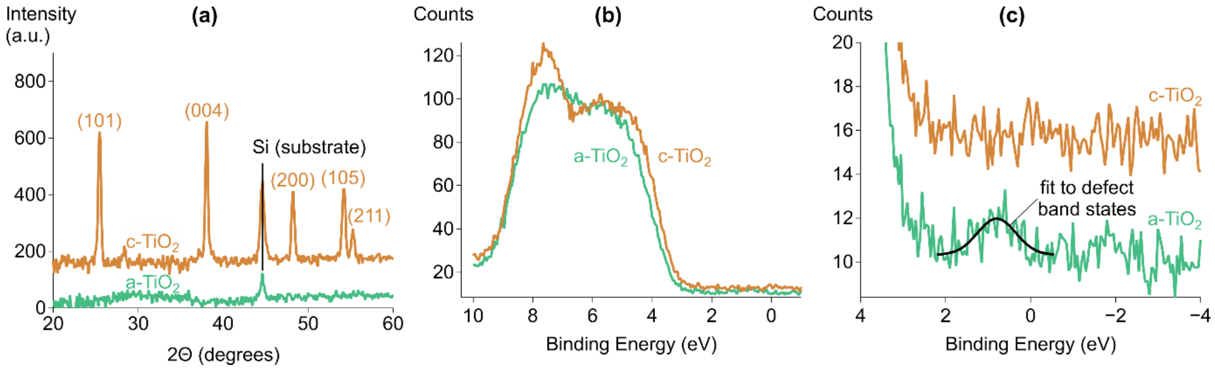

Figure S 8(a) XRD of a-TiO_2 and c-TiO_2. The c-TiO_2 peaks are indexed to PDF card 00-021-1272. (b) and (c) XPS data of a-TiO_2 and c-TiO_2.

Figure S8 shows the effects of crystallizing the a-TiO_2 film at 500°C. The annealed film is crystalline and has the anatase crystal structure, as expected with the annealing temperature. Also, as shown with XPS, the midgap defect band has been removed with the annealing treatment. This defect band has been associated with oxygen vacancies, or Ti^{3+} sites in the amorphous structure.[6] As the films are annealed in air, oxygen atoms are able to incorporate into the structure and remove the vacancies and therefore also the defect band.

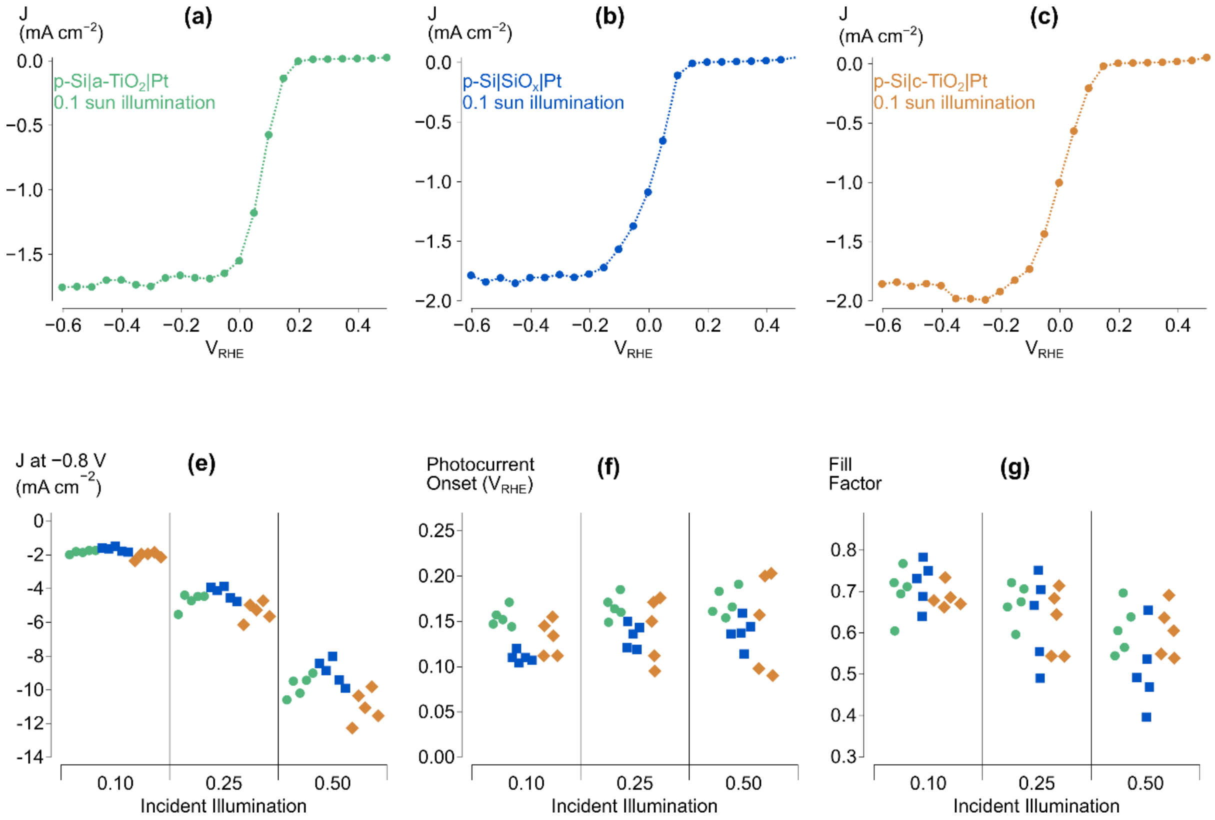

Figure S 9 (a), (b) and (c) Steady state current-potential curves of p-Si photocathode devices protected with a-TiO_2, SiO_x and c-TiO_2, respectively. (e), (f) and (g) Current density at −0.8 V vs RHE, onset voltages and fill factors of devices with different protection layers. Colors correspond to the protection layers as indicated in the graphs above.

Figure S9 compares three performance characteristics of devices with different protection layers. The current density at -0.8 V vs RHE is taken as the saturated photocurrent. For all devices, the photocurrent increases linearly with illumination intensity. The onset voltage normally increases with illumination intensity for each single device though significant sample to sample variation can be observed. The FF decreases for all devices upon increasing illumination intensity, with a larger effect on devices with the SiO_x protection layer (the FF was calculated using J_{sc} at −0.8 V vs RHE). Perhaps exposure of the oxide layer to the electrolyte increases the thickness beyond the native thickness of 1-2 nm and therefore reduces the tunnelling efficiency of photogenerated charges.

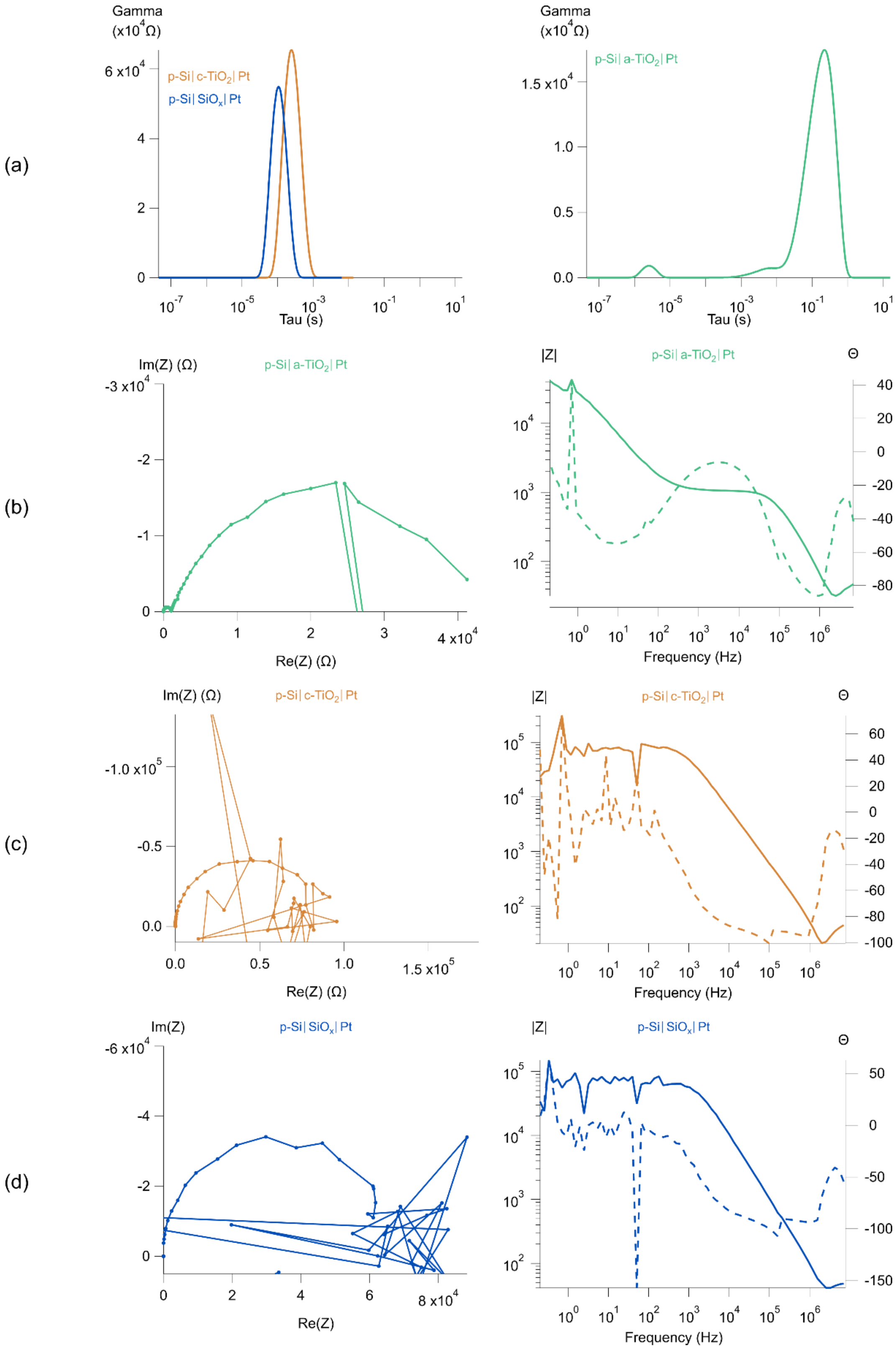

Figure S 10 (a) DRT analysis for samples, as indicated, at -0.55 mV vs RHE. (b) (c) and (d) corresponding Nyquist plots and Bode plots, showing raw experimental data. Noise at low frequencies, especially for the c-TiO_2 and SiO_x protected devices, limited the data that could be used for analysis. Note that some datapoints for the Nyquist plots are off the given scale.

Distribution of relaxation times (DRT) was performed using pyDRTtools (github.com/ciuccislab/pyDRTtools).[7] Parameter settings are included in the table below. All parameters correspond to the default values except for the Regularization Derivative, which was set to 2nd order. The same frequency range for DRT analysis was used for the Nyquist plots shown in the main text (Figure 3). This was because, as shown above in Figure S10, the noise in the data beyond these frequencies prevents fitting.

Method of Discretization	Gaussian
Data Used	Combined Re-Im Data
Inductance	Fitting w/o Inductance
Regularization Derivative	2nd order
Parameter Selection Method	Custom
Regularization parameter	0.001
Optimal Regularization Parameter	0.001
Number of S	1000

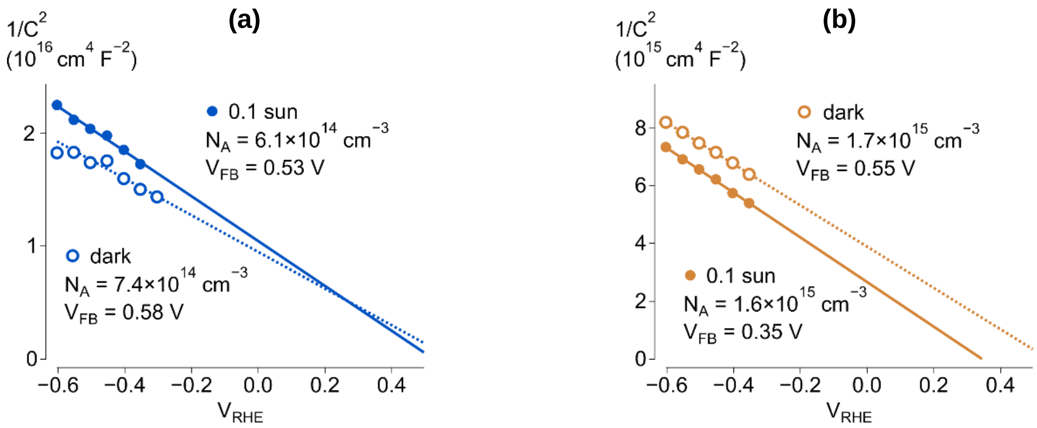

Figure S 11 Mott-Schottky plots of photocathode devices with (a) SiO_x and (b) c-TiO_2.

As the Nyquist plots of devices protected with either SiO_x or c-TiO_2 have only one semicircle, the data has been fit using the parallel RC circuit. The capacitor of this circuit has been used in Mott-Schottky plots to show that it is the depletion capacitance of silicon.

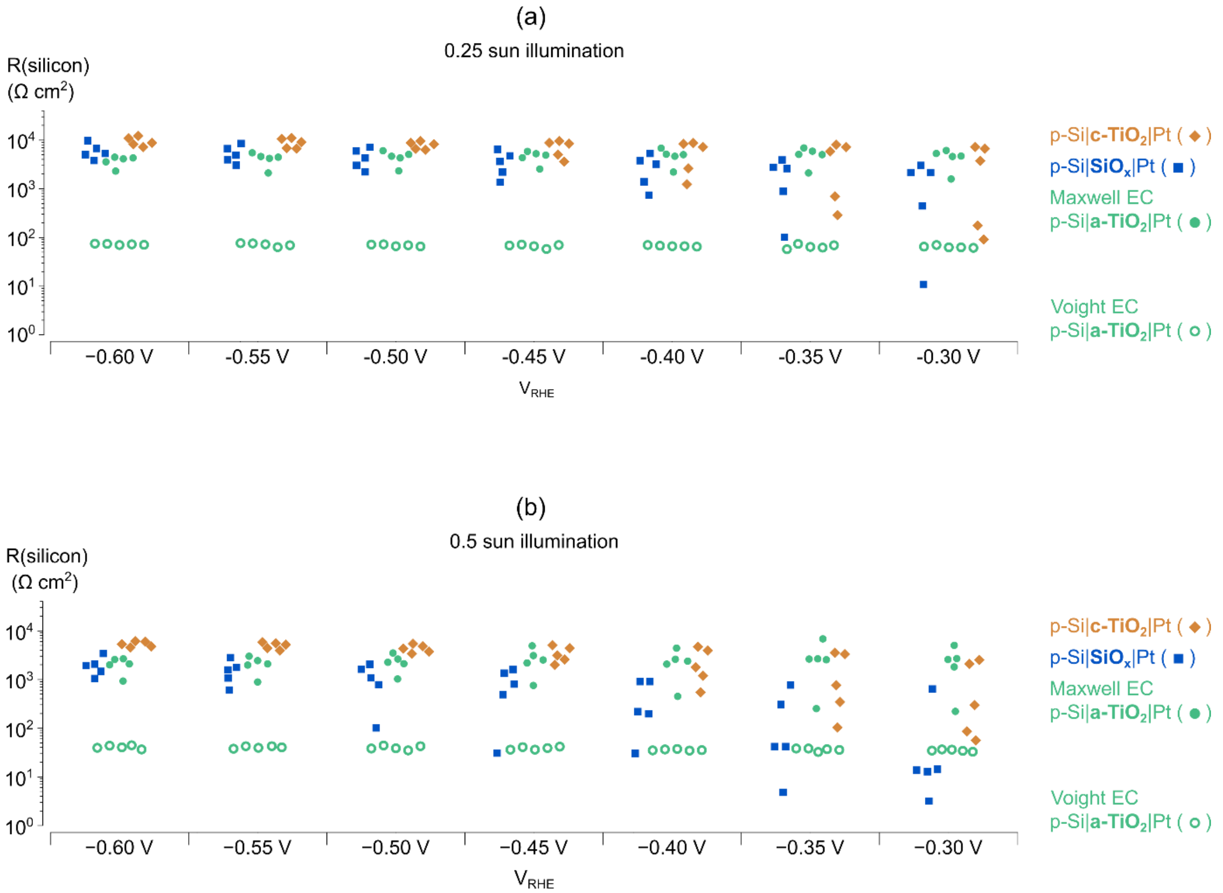

Figure S 12 Si resistance values in parallel with the depletion layer capacitance, extracted from EC analysis of devices operating under (a) 0.25 and (b) 0.5 sun illumination. For devices protected with a-TiO_2, two datasets are shown which correspond to each of the possible ECs, showing that the Maxwell circuit parameters closely match those of the other device types.

Under higher illumination intensities, the FF decreases slightly for all devices. Therefore they have not reached the saturated photocurrent at -0.3 V vs RHE, which is reflected in the observed decrease in the Si resistance at less negative potentials, when compared with the corresponding 0.1 sun experiment. However, at potentials where all devices have reached the saturation current, and the SiO_x and c-TiO_2 protected devices have unambiguous equivalent circuits, it is clear that the a-TiO_2 protected devices should again be modelled with the Maxwell circuit at these higher illumination intensities.

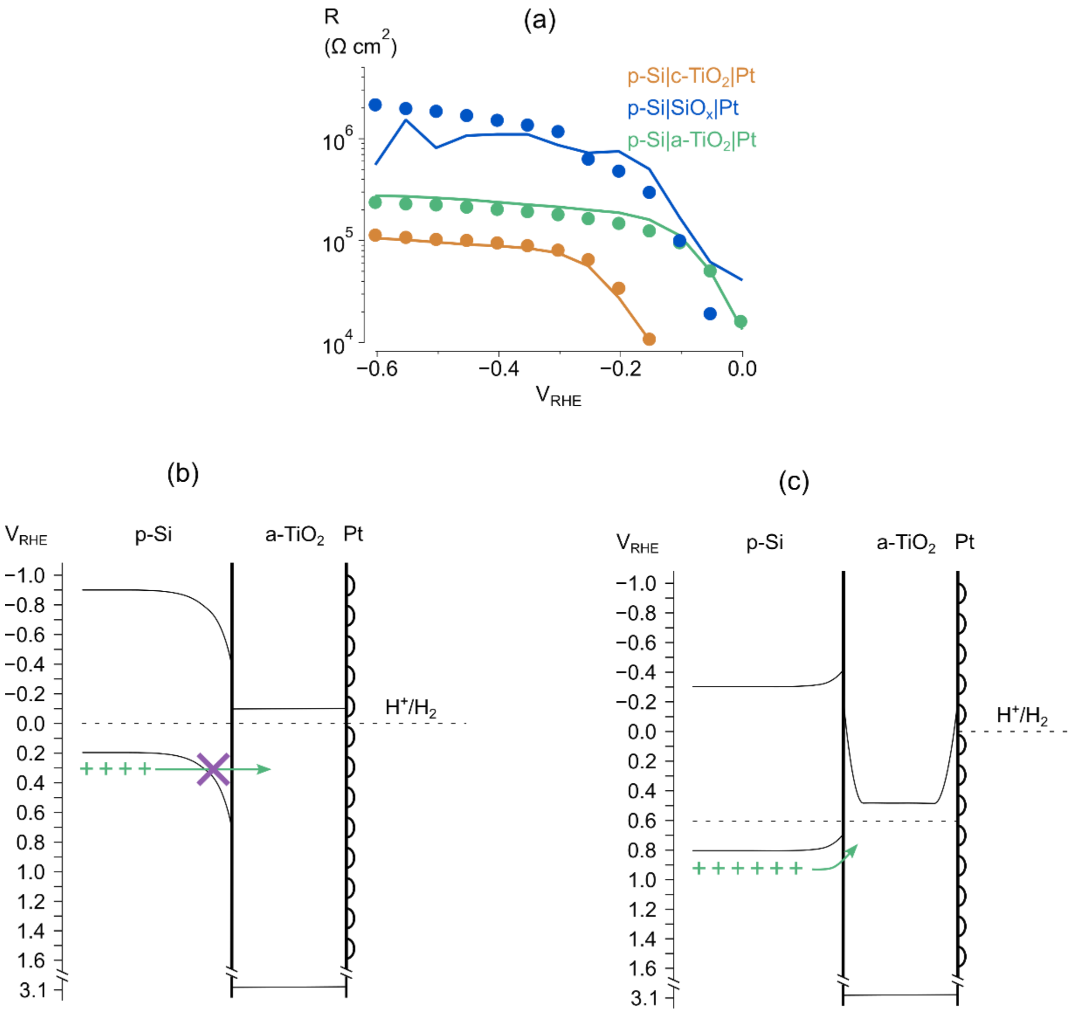

Figure S 13 (a) Resistances obtained from EIS (in markers) and from the slope of the current-voltage curve (in lines) in dark conditions. (b) Band diagram of an a-TiO_2 protected device, in the dark, at a potential where there is a large barrier in the p-Si for majority carriers to pass through the junction. This leads to a large Si resistance during EIS. (c) Band diagram of an a-TiO_2 protected device, in the dark, at a potential where there is no barrier in the p-Si for majority carriers. This leads to a small Si resistance during EIS.

To form the band diagrams in Figure S13 (b) and (c), we used the experimentally determined flat band potentials and estimated the energetic distance of the fermi level to the band considering the doping level (TiO_2 is highly doped, Si less doped). With the known bandgap of Si, this determines the conduction band offset. The conduction band offset was kept constant for all applied potentials. In Figure S11 (b), the band diagram looks similar to a photovoltaic solid-state device as the fermi level is set at the flatband potential of TiO_2. However, the electrolyte solution can differ from a metallic contact since the charge available for injecting into the TiO_2 is finite. At applied potentials positive of the hydrogen evolution reaction, the H_2 in solution is apparently not sufficient to maintain a split fermi level between the TiO_2 and the Si (as one would see in a 2-electrode device under applied potential): with applied potentials between ~ 0 and +0.4 V vs. RHE, we can observe the capacitance of the TiO_2 consistent with band bending at the TiO_2/electrolyte interface. We therefore draw a common fermi level between the Si and TiO_2 for all applied potentials.

For simplicity, we do not allow the band edges to slip at the Si/TiO_2 interface (which would reduce the band bending of the TiO_2 at the Si interface), although this likely occurs. Nevertheless, in such a case, the interpretation of the data regarding the equivalent circuit is not affected.

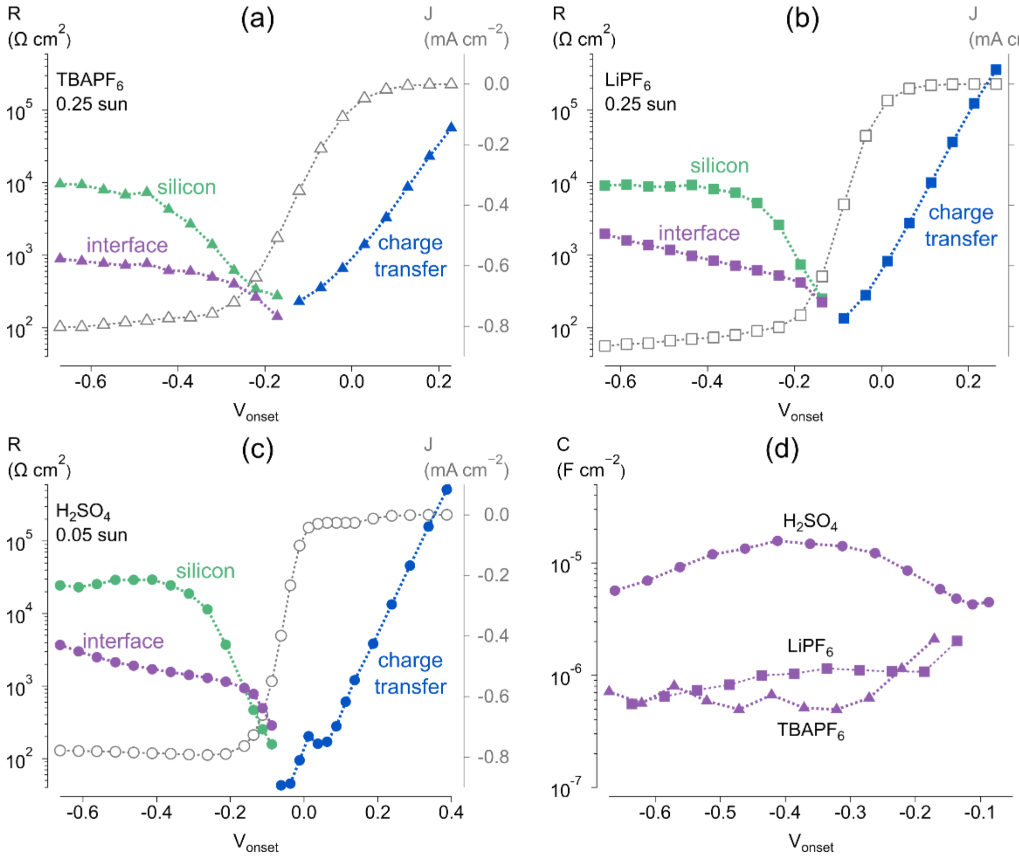

Figure S 14 EIS results of a-TiO_2 protected devices in (a) acetonitrile with $TBAPF_6$ supporting electrolyte, (b) acetonitrile with $LiPF_6$ supporting electrolyte and (c) 1 M H_2SO_4. (d) Comparison of the low frequency capacitances observed when using different supporting electrolytes.

As the ferrocenium ion absorbs strongly in the visible region, significant light attenuation occurred during the EIS experiments in aprotic solvent. For a direct comparison to the protic results, an experiment under 0.05 sun in 1 M H_2SO_4 was carried out. In all cases, a low frequency process in addition to the silicon RC element was observed. Therefore, we currently conclude that species intercalated into the a-TiO_2 are not the root cause of the non-faradaic process observed in p-Si|a-TiO_2|Pt devices.

Note that for the aprotic tests, a catalyst was not deposited as there is no significant overpotential associated with ferrocenium reduction on the surface of TiO_2.

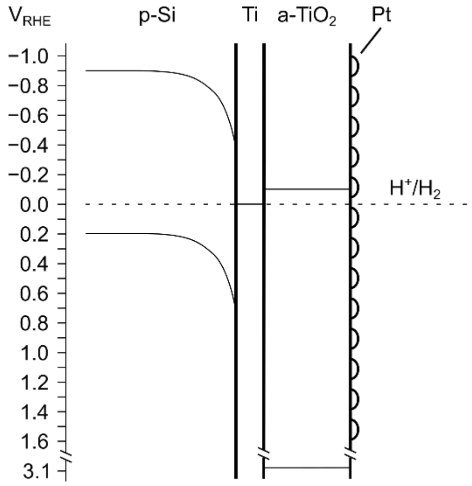

Figure S 15 Band diagram for a p-Si│Ti│a-TiO2│Pt device biased at 0 V vs RHE.

The p-Si│Ti│a-TiO_2│Pt device can be thought of as a buried Shottky junction, formed by the p-Si and Ti interface. Due to the Ti work function (4.33 eV in vacuum[8]) band bending in the p-Si evolves as it does for the p-Si│a-TiO_2 junction. The a-TiO_2 then forms an ohmic contact with Ti, due to the similarities in work function of these two materials. With the addition of Pt, the device is able to function as a PEC, although the incident light intensity on the p-Si is severely decreased due to the Ti layer. This is reflected in a low current density.

References

1. Yang, W., Moehl, T., Service, E. & Tilley, S. D. Operando Analysis of Semiconductor Junctions in Multi-Layered Photocathodes for Solar Water Splitting by Impedance Spectroscopy. *Adv. Energy Mater.* **11**, 2003569 (2021).

2. Busani, T. & Devine, R. A. B. Dielectric and infrared properties of TiO2 films containing anatase and rutile. *Semicond. Sci. Technol.* **20**, 870–875 (2005).

3. Dufond, M. E. *et al.* Quantifying the Extent of Ligand Incorporation and the Effect on Properties of TiO2 Thin Films Grown by Atomic Layer Deposition Using an Alkoxide or an Alkylamide. *Chem. Mater.* **32**, 1393–1407 (2020).

4. Patel, M. Y., Mortelliti, M. J. & Dempsey, J. L. A compendium and meta-analysis of flatband potentials for TiO2, ZnO, and SnO2 semiconductors in aqueous media. *Chem. Phys. Rev.* **3**, 011303 (2022).

5. Moehl, T., Suh, J., Sévery, L., Wick-Joliat, R. & Tilley, S. D. Investigation of (Leaky) ALD TiO2 Protection Layers for Water-Splitting Photoelectrodes. *ACS Appl. Mater. Interfaces* **9**, 43614–43622 (2017).

6. Nunez, P. *et al.* Characterization of Electronic Transport through Amorphous TiO2 Produced by Atomic Layer Deposition. *J. Phys. Chem. C* **123**, 20116–20129 (2019).

7. Wan, T. H., Saccoccio, M., Chen, C. & Ciucci, F. Influence of the Discretization Methods on the Distribution of Relaxation Times Deconvolution: Implementing Radial Basis Functions with DRTtools. *Electrochim. Acta* **184**, 483–499 (2015).

8. Neaman, D. A. *Semiconductor Physics and Devices: Basic Principles*. (McGraw-Hill, 2012).
